# Factors Influencing Adherence to Therapy With Occlusal Splints—A Multicentre Questionnaire Based Study

**DOI:** 10.1111/joor.70023

**Published:** 2025-07-24

**Authors:** Clara Christina Beck, Daniel Ralph Reissmann, Lisa Brinkmann, Oliver Schierz

**Affiliations:** ^1^ Department of Prosthodontics and Materials Science University of Leipzig Leipzig Germany; ^2^ Department of Prosthodontics and Materials Science University of Rostock Rostock Germany

**Keywords:** adherence, bruxism, dentistry, prospective human trial, stabilisation splints, temporomandibular disorders

## Abstract

**Background:**

Occlusal splints are an established treatment option for temporomandibular disorders (TMD) and for preventing the consequences of bruxism. However, the effectiveness of this therapy relies on the patient's adherence influenced by various factors. Understanding these factors is essential for improving adherence.

**Objectives:**

This study aimed to identify factors that promote and inhibit adherence to occlusal splint therapy in adult patients with TMD and/or bruxism, 4–8 weeks after insertion.

**Methods:**

Between July 2021 and December 2023, questionnaires were sent to adult patients who had received a splint within the previous 4 weeks. Of the 275 patients initially contacted, 200 completed the questionnaires. Adherence was self‐reported as the number of days the splint was worn per week and classified as high if worn at least 5 days or if dentists' recommendations were followed. Statistical analyses included the Chi‐square test, Fisher's exact test, the Mann–Whitney *U* test, and logistic regression to identify significant influencing factors.

**Results:**

67% of the enrolled patients showed high adherence. Three factors were positively associated with adherence: a positive attitude towards splint therapy (OR = 1.6), perceived positive treatment effects (OR = 2.5) and regulated sleeping times (OR = 3.0). In contrast, lower adherence was associated with sleep impairment (OR = 0.3), respiratory impairment (OR = 0.1) and increased salivation (OR = 0.3).

**Conclusion:**

Factors influencing adherence to occlusal splint therapy were identified during the critical early treatment phase. Patient‐centred strategies offer promising approaches to improve adherence to splint therapy. Further research is needed to confirm these findings, explore causal relationships and develop targeted interventions.

## Graphical abstract

Brief graphical summary with the main study results.

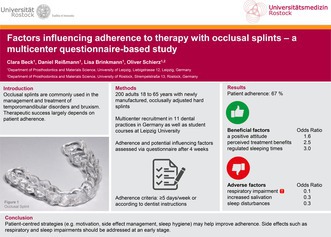

## 1 Background

Temporomandibular disorders (TMD) are considered the most common non‐odontogenic orofacial pain disorders [1], affecting approximately 34% of the global population [2]. Women are more affected than men, particularly in South America [2]. The global co‐occurrence of TMD and bruxism is estimated at 17%, with considerable regional variation, ranging from 70% in North America to just 9% in Asia [3]. The resulting reduction in patients' quality of life [4, 5] particularly oral health‐related quality of life (OHRQoL) [6], emphasises the relevance of effective treatment. A multidisciplinary approach often proves beneficial [7, 8]. More than 80% of patients can be treated satisfactorily with non‐surgical treatments [7]. Non‐invasive therapies include patient education, self‐help guidance, pharmacotherapy, topical ointment application, physiotherapy, behavioural therapy and occlusal appliances [9].

Hard occlusal splints represent one of the most commonly used types of occlusal appliances, with studies confirming their effectiveness in reducing pain and improving mandibular mobility [10, 11, 12]. A common characteristic of these splints is the provision of uniform occlusal contact as well as a canine‐guided dynamic occlusion. Neuromuscular models are increasingly applied to explain the effects of occlusal splints, although the underlying mechanisms are still being investigated. It is suggested that splints may enhance patients' cognitive awareness of their oral parafunctional habits [13]. Altered proprioception might lead to a modulation of muscular activity mediated by specific brain areas [14]. By reducing abnormal muscle activity, splints can help improve masticatory muscle function. Furthermore, it is proposed that splint use may facilitate neuroplastic recovery in affected brain regions, potentially contributing to symptom improvement [13]. Another potentially relevant mechanism is the reduction of intra‐articular stress through splint therapy [15]. Nevertheless, splints are only one therapeutic approach in current multimodal treatment concepts [16, 17]. The long‐term effectiveness of occlusal splint therapy remains a subject of debate, as some studies indicate no clear advantage over alternative TMD treatment approaches [18, 19, 20]. However, the objectives of splint therapy extend beyond the treatment of TMD, aiming also to protect dental hard tissues and reduce parafunctional activities such as bruxism [21, 22]. Its success largely depends on the patient's adherence. Although often used interchangeably, the term adherence emphasises a patient's active involvement, while compliance refers to simply following prescribed treatment. Adherence implies the patient's agreement, fostering more collaborative and less hierarchical communication. However, it is not only the actors who determine adherence. Many factors exert their influence [23].

Initial understanding of patient adherence to splint therapy has been gained through existing studies, which identified factors associated with high long‐term adherence to splint therapy, including a perceived good treatment effect [24], the treatment of long‐term conditions [24], and specific pain characteristics [25]. Furthermore, these studies highlighted patient‐reported barriers to splint use, such as comfort issues, disrupted sleep, solving the problem, perceived ineffectiveness, or increased pain [24, 25]. Lindfors et al. analysed adherence after a period of 1.5–2 years based on the number of days worn per week (high adherence ≥ 5 days) [24]. After this extended period, the proportion of patients with high adherence was still 52%. This aligns with findings from the long‐term study by Almoznino et al., which assessed adherence 1–8 years after splint insertion and categorised a wearing period of more than 1 year as adherent [25]. In this study, most patients (68.4%) who discontinued splint use stated that they had already stopped wearing it within 1 month.

Despite valuable insights from previous studies, significant research gaps remain. Considering early discontinuation, a better understanding of adherence in the early phase after splint insertion is crucial. Existing studies focus on long‐term adherence and self‐reported barriers to splint use or isolated associations between factors and adherence without providing a structured analysis. We hypothesised that associations between adherence and potential socioeconomic, patient‐related, health system‐related, condition‐related, and/or therapy‐related factors could be identified through statistical analysis, regardless of patients' subjective awareness of these connections.

This study aimed to identify factors that promote and inhibit adherence to occlusal splint therapy in adult patients with TMD and/or bruxism, 4–8 weeks after insertion.

## 2 Methods

### 2.1 Study Participants

**FIGURE 1:**
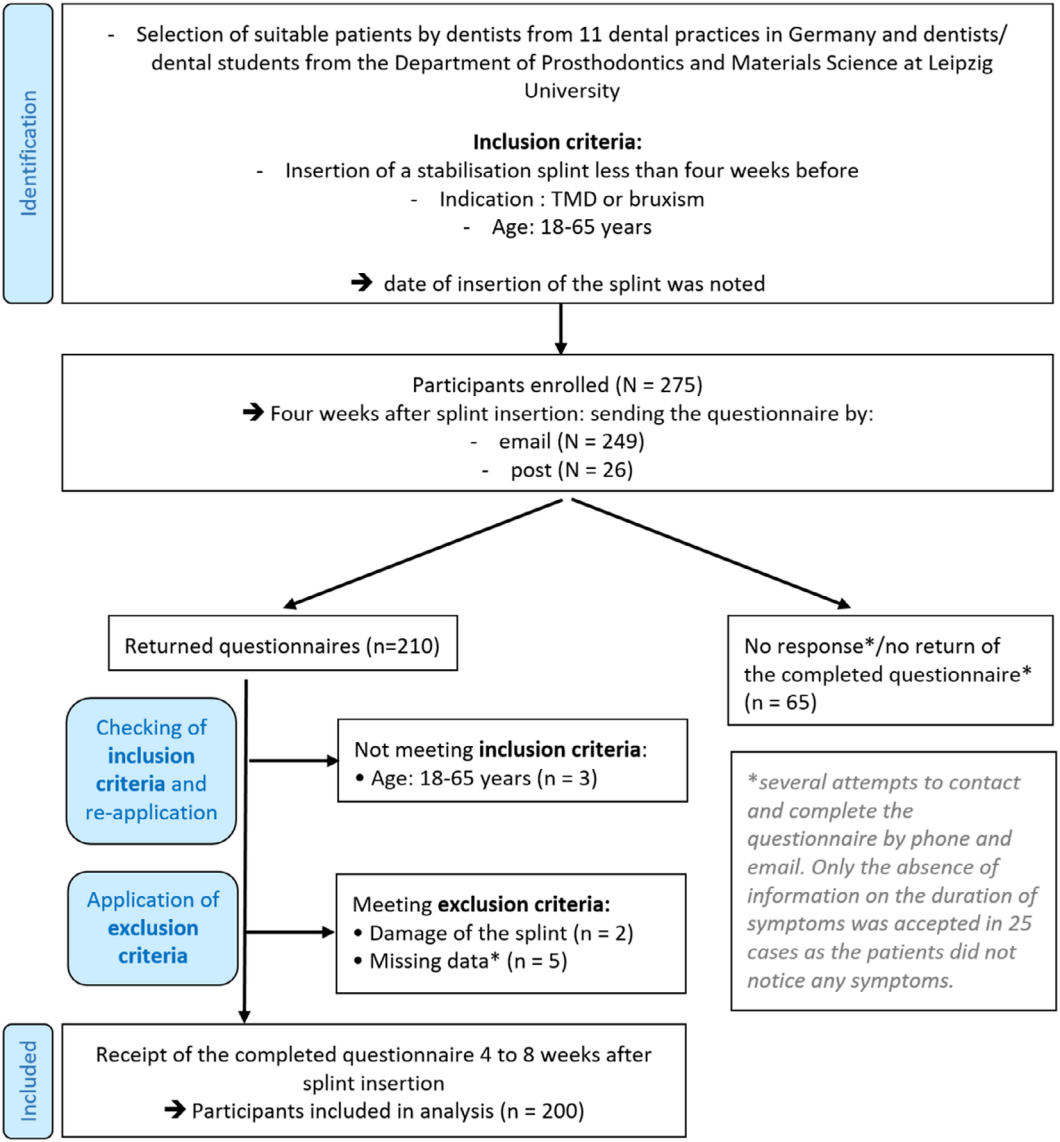
STROBE flow chart.

From July 2021 to December 2023, a total of 275 patients were selected, informed about the study details, and asked to participate by dentists from 11 dental practices in Germany (in Rhineland‐Palatinate, Saxony, North Rhine‐Westphalia, Berlin, Saarland) and by dentists and dental students from the Department of Prosthodontics and Materials Science at Leipzig University. The inclusion criteria were as follows: insertion of an occlusally adjusted splint in the upper jaw (UJ) or lower jaw (LJ) < 4 weeks prior, age between 18 and 65 years, and as an indication for splint therapy: TMD or bruxism. The diagnoses were made by the dentists based on clinical examination and medical history. This age range was chosen to focus on adults, excluding children, adolescents and seniors. The splint had to be made from a hard material, but no specifications were made regarding the specific design of the splints (e.g., thickness), material, or manufacturing process. The date of splint insertion was recorded, and written consent was obtained for future contact. Four weeks after splint insertion, an information sheet and a questionnaire (see Data S1) were either mailed (*n* = 249) as a fillable Portable Document Format (PDF) or sent in paper form (*n* = 26). Out of the 275 patients, 65 did not respond at all or failed to return the questionnaire, even after multiple follow‐up reminders (Figure 1). The refusal rate was 23.6%. A total of 210 patients returned the questionnaire, resulting in a response rate of approximately 76.4%. Among the patients who responded and returned the questionnaire, the inclusion criteria for age and indication were re‐checked by the study investigators to minimise potential sources of bias. Three patients did not fall within the age range (18–64 years). Two patients were excluded due to damage to the splint. Another five patients were excluded because they failed to fully complete their questionnaires, despite repeated follow‐up attempts. Missing data were supplemented in some cases through telephone or email communication. Only the absence of information on the duration of symptoms was accepted in 25 patients as they did not notice any symptoms. The remaining 200 patients were included in the study. The intended time frame for completing the questionnaire was between 4 and 8 weeks following splint insertion. In certain instances, multiple reminders were necessary, leading to questionnaire submissions delayed beyond 8 weeks after insertion.

The sample size was based on an a priori power analysis conducted with STATA version 15.1, assuming a two‐sided chi‐square test for comparing proportions (e.g., 20% vs. 40%), a significance level of *α* = 0.05, and a power of 80%. The required sample size for detecting a 20 percentage point difference between two groups was estimated at 164 participants (82 per group). In the case of smaller differences, such as a 10 percentage point difference, 300 participants per group would have been required to detect the differences. As a sample size of 300 participants per group was not feasible, the decision was made to include a total of 200 participants, exceeding the minimum requirement of 164, to ensure an adequate number of events per predictor for the logistic regression. This adjustment aimed to maintain statistical power and allow for stable and reliable estimation of the regression coefficients.

The present study was conducted in accordance with ethical standards and received the approval of the Ethics Committee on 6 July 2021 under file number 287/21‐ek.

### 2.2 Adherence Criteria

Adherence was determined based on the average number of days per week the splint was worn in the last month. Following the approach of Lindfors et al., patients who reported wearing the splint at least 5 days per week were classified as adherent (first adherence criterion) [24]. However, in our study, patients were also classified as adherent if they followed the wearing behaviour recommended by the dentist, despite a lower number of days per week on which the splint was worn. This included implementation of the recommendation for alternate wearing; adherence to the recommended number of days on which the splint should be worn; and realisation of the recommendation for demand‐oriented wearing (second, third and fourth adherence criterion).

### 2.3 Adherence Factors

Initially, a preliminary version of a questionnaire was designed based on the study by Lindfors et al. to investigate potential adherence factors [24]. This questionnaire was subsequently evaluated and refined using the ‘think aloud’ method with 10 participants between 11 February 2021 and 15 February 2021. The potential adherence factors identified in this process were structured around the five dimensions of adherence defined by the WHO (World Health Organisation), encompassing socioeconomic factors, patient‐related factors, health system‐related factors, condition‐related factors and therapy‐related factors [23]. At least three possible (non‐) adherence factors from all five WHO dimensions were considered. Most of the potential adherence factors were assessed using a 4‐point Likert scale (strongly agree, somewhat agree, somewhat disagree, strongly disagree). Multiple‐choice questions were used to gather information on variables such as gender, highest level of school education, therapists consulted, indications for splint therapy, upper or lower jaw splint, wearing behaviour and recommendations on wearing behaviour. Numerical response formats were applied for variables including age, duration of symptoms in months and the number of previous splints. Additionally, the severity of pain in the facial area was measured using a numeric rating scale ranging from 0 (no pain) to 10 (worst pain imaginable).

Some adherence factors were made up of several variables (see Tables 1 and 2 for details). Cronbach's alpha was calculated for the multivariable factors to assess internal consistency, with a value between 0.7 and 0.9 considered acceptable [27]. Based on these results, a total value was generated for factors within this range, reflecting reliable internal coherence. For factors with alpha values outside this range, individual variables were analysed separately to ensure a more precise interpretation. Additionally, the OHIP‐14 questionnaire was utilised as a validated instrument to measure oral health‐related quality of life [26, 28]. Its established scoring system involves summarising responses to 14 items, each rated on a Likert scale, providing a total score where higher values indicate a more significant impact on quality of life.

**TABLE 1:** Multivariable adherence factors and their composition.

Adherence factors	Composition of the adherence factors
Oral health‐related quality of life (OHRQoL)	14 variables: German version of the Oral Health Impact Profile (OHIP‐14) [26]; rating 0–4 for each item; sum score range 0–56
Positive attitude towards splint therapy	3 variables: conviction that the splint would help; conviction that wearing the splint would be useful; intention to wear the splint frequently Rating 0–3 for each item; Cronbach's *α* = 0.80; formation of a total value (range 0–9) [27]
Dissatisfaction with the splint	7 variables: unpleasant taste; too thick/uncomfortable shape; poor aesthetics; undesirable material; poor fit; defects; unhygienic Rating 0–3 for each item; Cronbach's *α* = 0.71, formation of a total value (range 0–21) [27]
Side effects	7 variables: speech impairment; respiratory impairment; dry mouth; increased salivation; halitosis; sleep impairment; discomfort Four‐point Likert scale for each item; Cronbach's *α* = 0.66, no total value formed [27]

**TABLE 2:** Description of the sample and results of the bivariate analysis of adherence factors and adherence.

Potential adherence factors divided into WHO dimensions	% (n) mean value (SD)^a^	Min–Max	Low adherence % (n) mean value (SD)^a^	High adherence % (n) mean value (SD)^a^	*p* ^b^
*Socio‐economic factors*					
Age in years	32.59 (11.68)	18–64	31.20 (11.62)	33.27 (11.70)	0.189
Gender					
Male	37% (74)		11.5% (23)	25.5% (51)	0.658
Female	63% (126)		21.5% (43)	41.5% (83)	
Highest level of school education (summarised)					
Without a General School Leaving Certificate or still in school or Secondary School Leaving Certificate or Intermediate School Leaving Certificate or equivalent qualification	13.5% (27)		4% (8)	9.5% (19)	0.689
Advanced Technical College Entrance Qualification or General University Entrance Qualification	86.5% (173)		29% (58)	57.5% (115)	
*Patient‐related factors*					
OHRQoL (OHIP‐14 sum score)	6.66 (6.86)	0–33	7.29 (7.31)	6.34 (6.63)	0.325
Stress during the last month					
No/little stress	34.5% (69)		8% (16)	26.5% (53)	**0.032**
Stress	65.5% (131)		25% (50)	40.5% (81)	
Positive attitude towards splint therapy—Sum score 0 to 9	7.40 (1.63)	1–9	6.42 (1.88)	7.89 (1.23)	**< 0.001**
Feeling of shame when wearing the splint in front of other people					
No/hardly any shame	70.5% (141)		20% (40)	50.5% (101)	**0.031**
Feeling of shame	29.5% (59)		13% (26)	16.5% (33)	
Perception of a positive treatment effect					
No	26.5% (53)		16% (32)	10.5% (21)	**< 0.001**
Yes	73.5% (147)		17% (34)	56.5% (113)	
Complaints worsen due to the splint					
No	7		6	1	
Yes	4		3	1	
Regulated sleeping times					
No	29% (58)		14.5% (29)	14.5% (29)	**0.001**
Yes	71% (142)		18.5% (37)	52.5% (105)	
*Health system‐related factors*					
Trust in the dentist					
No	1.5% (3)		1% (2)	0.5% (1)	0.211
Yes	98.5% (197)		32% (64)	66.5% (133)	
Good education about splint therapy					
Nein	5% (10)		2.5% (5)	2.5% (5)	0.303
Ja	95% (190)		30.5% (61)	64.5% (129)	
Good education about cleaning/care of the splint					
No	2.5% (5)		0.5% (1)	2% (4)	1.000
Yes	97.5% (195)		32.5% (65)	65% (130)	
Number of therapists consulted	0.82 (1.02)	0–4	0.82 (0.96)	0.81 (1.05)	0.693
*Condition‐related factors*					
Indications for splint therapy (summarised)					
Pain	38.5% (77)		14.5% (29)	24% (48)	0.488
Protecting my teeth	50% (100)		15.5% (31)	34.5% (69)	
Restriction of movement or unknown or other reasons	11.5% (23)		3% (6)	8.5% (17)	
Duration of symptoms in months	33.11 (38.74)	1–252	22.81 (26.89)	38.08 (42.54)	**0.003**
Severity of pain in the facial area last week (numerical rating scale 1–10)	1.46 (1.82)	0–9	1.48 (1.92)	1.44 (1.77)	0.970
Severity of pain in the facial area before the start of splint therapy (numerical rating scale 1–10)	2.91 (2.56)	0–9	3.02 (2.65)	2.85 (2.51)	0.721
*Therapy‐related factors*					
Number of previous splints	0.86 (1.44)		0.39 (0.68)	1.09 (1.65)	**0.004**
Upper jaw (UJ) or Lower Jaw (LJ) splint					
UJ	63% (126)		21% (42)	42% (84)	0.896
LJ	37% (74)		12% (24)	25% (50)	
Dissatisfaction with the splint—Sum score 0–21	3.58 (3.15)	0–15	4.97 (3.75)	2.90 (2.55)	**< 0.001**
Side effects					
Speech impairment	60.5% (121)		39% (78)	21.5% (43)	0.345
Respiratory impairment	6% (12)		5% (10)	1% (2)	**< 0.001**
Dry mouth	14% (28)		5.5% (11)	8.5% (17)	0.446
Increased salivation	40.5% (81)		19.5% (39)	21% (42)	**< 0.001**
Halitosis	9% (18)		4.5% (9)	4.5% (9)	0.108
Sleep impairment	17.5% (35)		12.5% (25)	5% (10)	**< 0.001**
Discomfort	11.5% (23)		8.5% (17)	3% (6)	**< 0.001**
Wearing behaviour: while sleeping and/or during the day					
Only while sleeping	95.5% (191)		30% (60)	65.5% (131)	0.062
Only during the day or when sleeping and/or during the day	4.5% (9)		3% (6)	1.5% (3)	
Recommendation on wearing behaviour: while sleeping and/or during the day					
Only while sleeping	82% (164)		25% (50)	57% (114)	0.107
Only during the day or when sleeping and/or during the day or no recommendation	18% (36)		8% (16)	10% (20)	
Recommendation on wearing behaviour: frequency					
Every day and/or night	81% (162)		27% (54)	54% (108)	0.836
Alternating on a weekly basis or a few times a week or as required or no recommendation	19% (38)		6% (12)	13% (26)	

*Note: p*‐values in bold: at significance (*p* < 0.05).

^a^ For metric variables, specification of mean values and standard deviation (SD). For categorical/ordinal variables, specification of absolute and relative frequencies.

^b^ For categorical variables based on the chi‐square test; for 2 × 2 cross‐tabulation with cells with an expected frequency of < 5: calculated with Fisher's exact test; for metric variables based on Mann–Whitney *U* test.

### 2.4 Data Analysis

The data were analysed using IBM SPSS Statistics version 29.0.1.1 (Armonk, NY: IBM Corp). The frequencies of potential adherence factors were presented descriptively for patients with high and low adherence, and a bivariate analysis was performed in a first step. In the case of categorical variables, the possible adherence factors were tested for an association to adherence using the chi‐square test or Fisher's exact test, respectively. Ordinal variables were first dichotomised. Agreement (strongly agree and somewhat agree) was distinguished from disagreement (somewhat disagree and strongly disagree). The chi‐square test was also carried out. As there was no normal distribution for the metric variables (Shapiro–Wilk‐test), the Mann–Whitney *U* test was used to analyse differences between the adherence groups.

Subsequently, all adherence factors with a *p*‐value of < 0.05 in the bivariate analysis were entered into a binary logistic regression with forward selection (likelihood ratio). Due to missing data on symptom duration in 25 cases, this variable was excluded from the model. Odds ratios (OR), 95% confidence intervals and *p*‐values were calculated. A *p*‐value of < 0.05 was assumed to be statistically significant. The effect size Cohen's *f* ^2^ for the logistic regression was calculated [29].

## 3 Results

### 3.1 Patient Characteristics

Of the 200 patients included in the study, 37% (*n* = 74) were male and 63% (*n* = 126) were female (Table 2). The average age of the participants was 32.59 years. Regarding the highest level of school education, most patients (86.5%, *n* = 173) reported having a General University Entrance Qualification or Advanced Technical College Entrance Qualification. The patient population primarily consisted of individuals undergoing splint therapy for bruxism, with 50% (*n* = 100) citing tooth protection as their main indication for treatment. However, a considerable proportion of patients presented with TMD, as 38.5% (*n* = 77) reported pain as their primary reason for splint therapy, and 4.5% (*n* = 9) reported movement restrictions as their main indication. A high level of adherence to splint therapy was observed in 67% (*n* = 134) of the sample. Among these, 126 patients wore their splint on average at least 5 days per week, meeting the first adherence criterion. Furthermore, eight patients were classified as adherent despite wearing the splint < 5 days per week. These patients fulfilled at least one of the three other adherence criteria (implementation of the recommendation to alternate wearing; adherence to the recommendation of a certain number of wearing days; implementation of demand‐oriented wearing).

### 3.2 Comparison Between Patients With High and Low Adherence

There were no significant differences between the high and low adherence groups in terms of socioeconomic factors (Table 2). Among the patient‐related factors, the variables stress (*p* = 0.032) and feeling ashamed when wearing the splint in front of other people (*p* = 0.031) were significantly more pronounced in the low adherence group. In contrast, a positive attitude towards splint therapy (*p* < 0.001), a perceived positive treatment effect (*p* < 0.001), and regulated sleeping times (*p* = 0.001) were more common in the high adherence group. No significant associations were found between health system‐related factors and adherence. Within the dimension of condition‐related factors, a longer duration of symptoms was observed more frequently in the high adherence group (*p* = 0.003). Twenty five participants reported no self‐perceived symptoms and therefore could not answer this question. Among the treatment‐related factors, a higher number of previous splints (*p* = 0.004) was linked to high adherence, whereas a high level of dissatisfaction (*p* < 0.001; see Table S1 for the specific dissatisfaction variables and their frequencies) and the side effects of respiratory impairment, increased salivation, sleep impairment, and discomfort were found to be associated with low adherence (all *p* < 0.001; see Table S2 for additionally mentioned side effects).

### 3.3 Effect of Factors Potentially Affecting Adherence

The binary logistic regression with forward selection (likelihood ratio) included all 200 patients and was statistically significant, *χ* ^2^ (6) = 77.399, *p* < 0.001, with a good variance explanation [30] of Nagelkerke's *R* ^2^ = 0.447. The overall percentage of correct classifications was 81%. Of the 12 factors analysed, six were included in the final model and showed significant associations. Among these, three adherence factors and three non‐adherence factors emerged from the analysis (Table 3). A one‐unit increase in the total value of the positive attitude towards splint therapy was associated with 1.6 times higher odds of high adherence. For patients who perceived a positive treatment effect, the odds of high adherence were 2.5 times higher. Patients with regulated sleeping times showed 3 times higher odds of high adherence. In contrast, three splint‐related side effects were associated with significantly lower odds of high adherence. Patients who experienced respiratory impairment due to the splint had 90% lower odds of high adherence (OR = 0.1). Increased salivation and sleep impairment caused by the splint were both linked to 70% lower odds of high adherence (OR = 0.3 each). Cohen's *f* ^2^ was 0.81, which, according to Cohen [29] corresponds to a strong effect.

**TABLE 3:** Results of the logistic regression (*N* = 200).

Included factors	Odds ratio	95% confidence interval	*p*
Adherence factors			
Positive attitude towards splint therapy (total value)	1.6	1.2–2.2	0.001
Perception of a positive treatment effect	2.5	1.04–5.9	0.041
Regulated sleeping times	3.0	1.4–6.7	0.007
Non‐adherence factors			
Respiratory impairment	0.1	0.02–0.7	0.017
Increased salivation	0.3	0.2–0.7	0.003
Sleep impairment	0.3	0.1–0.9	0.023

## 4 Discussion

The study identified a high adherence rate to splint therapy, with 67% of patients meeting the adherence criteria defined as wearing their splints at least 5 days per week or following dentists' recommendations respectively. Three patient‐related factors showed to be associated with better adherence: a positive attitude towards splint therapy, positive treatment effect and regulated sleeping times. In contrast, side effects such as respiratory impairment, increased salivation and sleep impairment, which fall under the therapy‐related factors dimension, were linked to lower adherence.

A positive attitude towards splint therapy was associated with higher adherence (OR = 1.6), highlighting the importance of patient motivation and guidance. Most patients reported a positive treatment effect, which was linked to 2.5 times higher odds of high adherence. Lindfors et al. similarly found that long‐term splint users reported better outcomes, likely mediated by motivation [24]. Another study indicated higher adherence in patients who experienced more than 50% pain relief, except when pain was entirely eliminated, presumably due to the lack of need for the splint [25]. These findings support follow‐up care to reinforce treatment benefits early on. In the present study, only 4 patients (2%) reported a negative treatment effect, confirming Lindfors et al. who deemed this risk negligible [24]. Patients with regulated sleeping times had three times higher odds of high adherence. An association between sleep (quality and duration) and TMD has already been demonstrated, leading to recommendations for ensuring adequate sleep to manage TMD and orofacial pain better [31]. Regulated sleep may contribute to both improved adherence to splint therapy and reduced TMD symptoms. Given the availability of behavioural strategies to improve sleep hygiene, this could represent a good modifiable factor. However, it should be noted that patients with regular sleep patterns may also have higher general health awareness, which could independently support better adherence. In contrast, sleep impairment due to the splint was associated with a 70% reduction in the odds of high adherence (OR = 0.3). Almoznino et al. [25] and Lindfors et al. [24] similarly identified sleep disturbances as a key reason for discontinued splint use. In the present study, two further side effects were also significantly associated with low adherence. Respiratory impairment caused by the splint reduced the odds of high adherence by 90% (OR = 0.1), while increased salivation was linked to 70% lower odds of high adherence (OR = 0.3). In order to reduce the side effects, a compromise between a more comfortable design and the assurance of sufficient material for therapeutic effectiveness and long‐term durability should be found. Digital workflows offer advantages such as increased precision, accuracy, time efficiency and patient comfort [32]. However, conventional splints remain a durable option, particularly for long‐term use [33], and do not require investment in digital equipment [32]. The most common reason for dissatisfaction with the splint was its thickness/shape. Although not included in the regression model, the bivariate analysis showed an association for the dissatisfaction variable with low adherence. Previous research has demonstrated the efficacy of treating TMD with splints of varying thickness [34]. Current research underscores the need to identify the most suitable splint type for each patient, particularly by assessing adverse effects in future studies [35]. While this study provides initial insights for splints, further research is needed to guide individualised, evidence‐based treatment decisions. If the splint, despite efforts to ensure a comfortable design, still leads to sleep disturbances, it may be necessary to re‐evaluate whether alternative treatment options might be more appropriate. Given that impaired sleep quality has even been linked to reduced life satisfaction [36], the aspect of patient‐centred treatment approaches to suit the unique demands of each patient could be relevant both for selecting the type of splint [35] and for choosing a TMD therapy tailored to the individual patient. As previously discussed, other treatment approaches appear to be equally effective as occlusal splint therapy in the long term [18, 19, 20]. However, finding an alternative becomes problematic in cases of bruxism and resulting tooth wear. Other significant factors in the bivariate analysis, though not included in the regression model, were stress, shame about wearing the splint in front of others, number of previous splints and symptom duration.

While our findings largely align with previous research, no association between the patient‐reported indication and adherence was found in the present study. The most frequently reported indication was protection of the teeth, which can be interpreted as protection from the consequences of bruxism. Similarly, Lindfors et al. reported that the most common indication in general dental practices was tooth wear due to bruxism [24]. The authors demonstrated that, for specific indications that can be understood as long‐term conditions, adherence was higher after 1.5–2 years. In contrast, the study by Almoznino et al. found higher adherence among patients with painful diagnoses [25]. Notably, the low long‐term adherence in patients with sleep bruxism, a condition that is generally not painful, was striking, while painful diagnoses, such as masticatory muscle dysfunction, were associated with a higher proportion of patients continuing to use their splint. Both long‐term and painful conditions may explain increased adherence due to heightened distress. Although no associations between indication and adherence were found 4–8 weeks after splint insertion in the present study, self‐reported symptom duration was linked to adherence in the bivariate analysis. Future studies should establish whether long‐term conditions or painful diagnoses are more strongly associated with higher adherence in both the short and long term. Additionally, future research should aim to include clinically verified diagnoses reported by dental professionals, rather than self‐reported diagnoses. Furthermore, it would be interesting to investigate the influence of splint position (upper or lower jaw) and obstructive sleep apnoea symptoms on perceived sleep disturbances when wearing splints.

The findings of our study suggest the potential value of patient‐centred strategies to support adherence to splint therapy. Regulated sleeping times were associated with improved adherence, suggesting the potential benefit of incorporating sleep hygiene recommendations into treatment plans. Identifying potential barriers to adherence, such as side effects or motivational deficits, at an early stage may be beneficial. Regular follow‐up care could help address barriers and reinforce the perceived benefits of therapy. Furthermore, strategies to support adherence may include optimising splint design to balance therapeutic effectiveness and comfort, and addressing side effects such as respiratory or sleep disturbances. If the splint leads to adverse impacts despite efforts to ensure comfort, it is essential to reconsider the treatment approach. This includes not only selecting the most appropriate type of splint, but also evaluating whether a splint is indeed the most suitable therapeutic option for the individual patient. However, these findings should be interpreted with caution, as further research is needed to confirm the associations and develop targeted interventions.

Adherence is a key factor in the success of various dental treatments, yet more research is needed to understand it fully across disciplines. Studies highlight that elements like digital tools, caregiver support, and clinician relationships can enhance adherence, especially in orthodontics and mandibular advancement device therapy, while demographics seem less influential [37, 38]. In prosthodontics, educational interventions show promise, but broader, comparative research is still required [39].

Our study's key strength lies in its approach of specifically querying potential factors influencing adherence and analysing their associations with adherence outcomes rather than merely asking patients to identify what they believe impacts their adherence. This method allowed for a more systematic exploration of the actual factors, offering a clearer understanding of the relationships involved. A further strength of this study is its large sample size, which allowed for precise estimates, the high response rate and its multicentre approach, enhancing the representativeness and generalisability of the findings. These strengths ensure a large bandwidth of inter‐individual treatment concepts, offering high informative value and reducing patient‐ and concept‐based bias. The potential adherence factors were primarily derived from an existing adherence study and were subsequently enriched through qualitative feedback obtained from 10 participants. The WHO's five dimensions of adherence [23] were employed as an established framework to structure the factors. The combination of these methods ensured that a large number of potentially relevant factors were identified, drawing from various dimensions of adherence.

As a limitation of the study, this approach may not capture all context‐specific or less apparent factors influencing adherence. The potential factors identified in this study cannot be considered exhaustive. The manufacturing of the splints, including materials and devices, was not prescribed nor inquired about in the study. Therefore, no conclusions can be drawn regarding which splint design or material may lead to the mentioned side effects. Moreover, it must be noted that all information was based on self‐reported data. Due to the consecutive sampling, a distortion of the data compared to the general population cannot be ruled out. The homogeneous educational background may have introduced bias and limited the generalisability of the findings, as potential influences of other educational levels on adherence could not be examined. Furthermore, it is possible that patients with poorer adherence may have been less motivated to complete and return the questionnaires, which could also introduce bias. Additionally, unmeasured confounding factors, such as general health awareness, may have influenced both adherence and the reported predictors. The correlations found in this study do not imply causality.

## 5 Conclusion

Building upon prior long‐term research, this study identifies key factors influencing adherence to splint therapy during the crucial early phase of treatment, where the dropout rate appears highest. Four to eight weeks after insertion, high adherence was found to be associated with a positive attitude towards therapy, perceived treatment benefits and regulated sleep patterns. In contrast, side effects such as respiratory impairment, increased salivation and sleep disturbances were linked to lower adherence. By employing a large sample size, a two‐step statistical analysis, a multicentre design and a wide range of factors structured around the WHO's five dimensions of adherence [23], the study offers a comprehensive understanding of determinants of adherence. Further studies are necessary to validate these associations, explore causal relationships and develop tailored strategies for intervention.

## Conflicts of Interest

The authors declare no conflicts of interest.

## Supporting information


**Data S1.** Questionnaire.

**Table S1.** Dissatisfaction variables and their frequencies (*N* = 200).
**Table S2.** Rare mentioned side effects listed in alphabetic order.

## Data Availability

The data that support the findings of this study are available from the corresponding author [OS] upon reasonable request.
