# Supplementary material for: Factors Influencing Adherence to Therapy With Occlusal Splints—A Multicentre Questionnaire Based Study
Source: J Oral Rehabil. 2025 Jul 24;52(12):2209–18. doi: 10.1111/joor.70023 (PMC12624160; doi:10.1111/joor.70023)
Supplement: Supplementary file 1 — Data S1. Questionnaire. [file JOOR-52-2209-s001.pdf]

Patientenaufklärung zur Studie:

„Faktoren mit Einfluss auf die Adhärenz bei Therapie mit Äquilibrierungsschienen“

Sehr geehrte Patientin, sehr geehrter Patient,
mit diesem Schreiben laden wir Sie ein, an der oben genannten Studie teilzunehmen. Bitte lesen Sie sich folgende Informationen sorgfältig durch. Sie können dann entscheiden, ob Sie teilnehmen möchten oder nicht. Lassen Sie sich ausreichend Zeit.

Welches Ziel verfolgt die Studie?
Ziel dieser Studie ist die Erfassung von Faktoren, die einen Einfluss auf das Trageverhalten erwachsener Patienten bei Therapie mit Äquilibrierungsschienen haben. So soll zu einem besseren Verständnis der den Therapieerfolg mitbestimmenden Tragegewohnheiten beigetragen werden.

Was muss beachtet werden?
Voraussetzung für Ihre Teilnahme an der Studie ist, dass Sie **vor 4 Wochen eine Äquilibrierungsschiene für Ihren Ober- oder Unterkiefer** von Ihrem Zahnarzt/Ihrer Zahnärztin erhalten haben. Sie sollten das **18. Lebensjahr** vollendet haben und **nicht älter als 65 Jahre** alt sein. Wenn dies der Fall ist, sind Sie für diese Studie geeignet.
Falls Sie allerdings Ihre **Schiene verloren** haben oder diese so **starke Beschädigungen** aufweist, dass sie **nicht mehr tragbar** ist, können Sie an der Studie leider nicht teilnehmen. Ebenso sollten Sie die Schiene nicht bekommen haben, um eine **neue Bisslage** auszutesten.

Wie ist der Ablauf?
Bitte beantworten Sie alle Fragen vollständig und so, wie es Ihnen richtig erscheint. Sie können sich so viel Zeit nehmen, wie Sie brauchen. Versuchen Sie spontan und zügig zu antworten. Falls Sie bei einer Frage Zweifel haben, welche Antwort zutrifft, kreuzen Sie bitte nur die eine Antwort an, die am ehesten für Sie zutrifft.

Informationen zum Datenschutz
Die **ärztliche Schweigepflicht** und **datenschutzrechtliche Bestimmungen** werden eingehalten. Während der Studie werden persönliche Informationen von Ihnen erhoben. Die Auswertung der Patientendaten erfolgt pseudonymisiert. Die Daten sind gegen unbefugten Zugriff gesichert. Eine Entschlüsselung erfolgt nur bei Rücktritt von der Studie zum Zweck der Datenvernichtung. Sobald es nach dem Forschungs- oder Statistikzweck möglich ist, werden die personenbezogenen Daten anonymisiert, jedoch spätestens 6 Monate nach Abschluss der Studie. Die während der Studie erhobenen Daten werden nach Abschluss der Datenauswertung gemäß den gesetzlichen Vorschriften für 10 Jahre aufbewahrt. Die Daten werden ausschließlich zu Zwecken dieser Studie verwendet.
Sie haben das Recht, vom Verantwortlichen (s.u.) Auskunft über die von Ihnen gespeicherten personenbezogenen, pseudonymisierten Daten zu verlangen. Ebenfalls können Sie die Berichtigung unzutreffender Daten sowie die Löschung der Daten oder Einschränkung deren Verarbeitung verlangen.

Der Verantwortliche für die studienbedingte Erhebung personenbezogener Daten ist:
Universitätsklinikum Leipzig
Poliklinik für Zahnärztliche Prothetik und Werkstoffkunde
Priv.-Doz. Dr. Oliver Schierz
Liebigstr. 12
04103 Leipzig
Tel.: 0049 (0)97-21-310

Bei Anliegen zur Datenverarbeitung und zur Einhaltung der datenschutzrechtlichen Anforderungen der übermittelten pseudonymisierten Daten können Sie sich an folgenden Datenschutzbeauftragten der Einrichtung wenden:

Datenschutzbeauftragter der Medizinischen Fakultät
Philipp-Rosentalstr. 27, Haus M
04103 Leipzig
dsbmf@medizin.uni-leipzig.de

Datenschutzbeauftragter des Universitätsklinikums Leipzig
Liebigstr. 18, Haus B
04103 Leipzig
dsb@uniklinik-leipzig.de

Einverständnis
Mit der Rücksendung dieses Dokumentes (*„Patientenaufklärung und Fragebogen - Faktoren mit Einfluss auf die Adhärenz bei Therapie mit Äquilibrierungsschienen")* bestätigen Sie Ihre Kenntnisnahme der Patientenaufklärung sowie Ihr Einverständnis zur Speicherung und pseudonymen Bearbeitung der im Rahmen der Studie erhobenen Daten zur wissenschaftlichen Auswertung.

Freiwilligkeit/Rücktritt
Die Teilnahme an der Studie erfolgt freiwillig. Sie können Ihre Einwilligung jederzeit schriftlich oder mündlich ohne Angabe von Gründen widerrufen, ohne dass Ihnen dadurch Nachteile entstehen. Wenn Sie Ihre Einwilligung widerrufen möchten, wenden Sie sich bitte an die Studienleitung. Bei einem Widerruf können Sie entscheiden, ob die von Ihnen studienbedingt erhobenen Daten gelöscht werden sollen oder weiterhin für die Zwecke der Studie verwendet werden dürfen. Auch wenn Sie einer weiteren Verwendung zunächst zustimmen, können Sie nachträglich Ihre Meinung noch ändern und die Löschung der Daten verlangen. Wenden Sie sich dafür bitte ebenfalls an die Studienleitung. Beachten Sie, dass Daten, die bereits in wissenschaftliche Auswertungen eingeflossen sind oder Daten, die bereits anonymisiert wurden, nicht mehr auf Ihren Wunsch gelöscht / vernichtet werden können.

Weitere Informationen
Für weitere Informationen sowie für Auskünfte über allgemeine Ergebnisse und den Ausgang der Studie steht Ihnen an der Universität Leipzig als Leiter der Studie Herr Priv.-Doz. Dr. Oliver Schierz (Telefon: 0341-9721310, Email: oliver.schierz@medizin.uni-leipzig.de) zur Verfügung.

Für Ihre Teilnahme an diesem Forschungsprojekt wären wir Ihnen sehr dankbar!

UNIVERSITÄT LEIPZIG

Universitätsklinikum Leipzig
Medizin ist unsere Berufung.

Befragung zur Studie:

„Faktoren mit Einfluss auf die Adhärenz bei Therapie mit Äquilibrierungsschienen“

Patienten-ID:
Bitte schreiben Sie in die vorderen beiden Kästchen die **ersten beiden Anfangsbuchstaben** des **Vornamens Ihrer Mutter**, in Kästchen 3 und 4 die ersten beiden Anfangsbuchstaben des **Vornamens Ihres Vaters** und in die hinteren Kästchen **Ihr Geburtsjahr**.
Dies dient der Pseudonymisierung Ihrer Daten.

Bsp.:
Vorname der Mutter: **An**na
Vorname des Vaters: **Th**omas
Ihr Geburtsjahr: 19**90**
Beispiel-ID: **A N T H 9 0** **Ihre ID:**

Heutiges Datum: **Datum der Eingliederung der Schiene:**

**Wenn Sie an die letzten vier Wochen denken…<br>…an wie vielen Tagen PRO WOCHE haben Sie Ihre Schiene durchschnittlich getragen?**							
7 Tage	6 Tage	5 Tage	4 Tage	3 Tage	2 Tage	1 Tag	0 Tage

**Tragen Sie Ihre Schiene beim Schlafen und/oder tagsüber?**	
Ich trage die Schiene nur beim Schlafen.	○
Ich trage die Schiene nur tagsüber.	○
Ich trage die Schiene beim Schlafen <u>und</u> tagsüber.	○
Ich trage die Schiene beim Schlafen <u>oder</u> tagsüber.	○

In den folgenden Fragen geht es um die Stärke Ihrer Schmerzen im Gesichtsbereich. Sie können die Angaben jeweils auf einer Skala von 0 bis 10 abstufen. Der Wert 0 bedeutet, dass Sie keine Schmerzen haben/hatten, der Wert 10 bedeutet, dass die Schmerzen nicht schlimmer sein könnten. Mit den dazwischen liegenden Werten können Sie Abstufungen vornehmen.										
**Falls Sie <u>in der letzten Woche</u> unter Schmerzen im Gesichtsbereich litten, als wie stark empfanden Sie diese durchschnittlich?**										
0	1	2	3	4	5	6	7	8	9	10
**Falls Sie <u>vor Beginn der Schienentherapie</u> unter Schmerzen im Gesichtsbereich litten, wie stark empfanden Sie diese durchschnittlich?**										
0	1	2	3	4	5	6	7	8	9	10

**Hatten Sie im vergangenen Monat aufgrund von Problemen mit Ihren Zähnen, im Mundbereich oder mit Ihrem Zahnersatz…**	**sehr oft**	**oft**	**ab und zu**	**kaum**	**nie**
1. Schwierigkeiten, bestimmte Worte auszusprechen?	○	○	○	○	○
2. das Gefühl, Ihr Geschmackssinn war beeinträchtigt?	○	○	○	○	○
3. den Eindruck, dass Ihr Leben ganz allgemein weniger zufriedenstellend war?	○	○	○	○	○
4. Schwierigkeiten zu entspannen?	○	○	○	○	○
**Ist im vergangenen Monat aufgrund von Problemen mit Ihren Zähnen, im Mundbereich oder mit Ihrem Zahnersatz vorgekommen, …**	**sehr oft**	**oft**	**ab und zu**	**kaum**	**nie**
5. dass Sie sich angespannt gefühlt haben?	○	○	○	○	○
6. dass Sie Ihre Mahlzeiten unterbrechen mussten?	○	○	○	○	○
7. dass es Ihnen unangenehm war, bestimmte Nahrungsmittel zu essen?	○	○	○	○	○
8. dass Sie anderen Menschen gegenüber eher reizbar gewesen sind?	○	○	○	○	○
9. dass es Ihnen schwergefallen ist, Ihren alltäglichen Beschäftigungen nachzugehen?	○	○	○	○	○
10. dass Sie vollkommen unfähig waren, etwas zu tun?	○	○	○	○	○
11. dass Sie sich ein wenig verlegen gefühlt haben?	○	○	○	○	○
12. dass Ihre Ernährung unbefriedigend gewesen ist?	○	○	○	○	○
**Hatten Sie im vergangenen Monat…**	**sehr oft**	**oft**	**ab und zu**	**kaum**	**nie**
13. Schmerzen im Mundbereich?	○	○	○	○	○
14. ein Gefühl der Unsicherheit in Zusammenhang mit Ihren Zähnen, Ihrem Mund oder Ihrem Zahnersatz?	○	○	○	○	○

Auch die folgende Aussage bezieht sich auf die letzten vier Wochen. Bitte bewerten Sie diese anhand der vorgegebenen Auswahlmöglichkeiten.

	**Stimme völlig zu**	**Stimme eher zu**	**Stimme eher nicht zu**	**Stimme gar nicht zu**
Im vergangenen Monat habe ich mich häufig gestresst gefühlt.	○	○	○	○

In den folgenden Aussagen bewerten Sie bitte das Vertrauen in Ihre/n Zahnarzt/-ärztin und die Aufklärung und Pflegehinweise, die Sie durch ihn/sie oder das Praxisteam bezüglich der Schiene erfahren haben.

	**Stimme völlig zu**	**Stimme eher zu**	**Stimme eher nicht zu**	**Stimme gar nicht zu**
Ich habe Vertrauen in meine/n Zahnarzt/-ärztin und dessen/deren Fähigkeiten.	○	○	○	○
Mein/e Zahnarzt/-ärztin hat mich gut über die Behandlung mit der Schiene aufgeklärt und informiert.	○	○	○	○
Ich weiß, wie ich meine Schiene richtig pflege/säubere.	○	○	○	○

In den folgenden Aussagen geht es darum, wie Sie über Ihre Schiene denken.				
	Stimme völlig zu	Stimme eher zu	Stimme eher nicht zu	Stimme gar nicht zu
Ich bin davon überzeugt, dass die Schiene mir bei meinen Beschwerden helfen wird.	○	○	○	○
Es erscheint mir sinnvoll, dass ich die Schiene tragen soll.	○	○	○	○
Ich habe mir vorgenommen, die Schiene häufig zu tragen.	○	○	○	○
Es ist mir unangenehm, die Schiene im Beisein von anderen Personen zu tragen (z.B. PartnerIn/MitbewohnerIn/ Arbeitskollege/in).	○	○	○	○
Ich kann schon einen positiven Effekt durch das Tragen der Schiene spüren.	○	○	○	○
**FALLS Sie gar KEINEN positiven Effekt spüren, bewerten Sie bitte auch folgende Aussage:** Meine Beschwerden verschlimmern sich durch das Tragen der Schiene.				

Es folgt eine Aussage zu Ihrem Schlafverhalten.				
	Stimme völlig zu	Stimme eher zu	Stimme eher nicht zu	Stimme gar nicht zu
Ich gehe in der Regel ungefähr zur selben Uhrzeit ins Bett.	○	○	○	○

**Deswegen habe ich die Schiene bekommen (Bitte nur den Hauptgrund angeben.):**				
○ Schmerzen	○ Schutz meiner Zähne	○ Bewegungs-einschränkung (z.B. Mundöffnungs-einschränkung)	○ Weiß ich nicht.	○ Anderer Grund*

***Falls: *Anderer Grund* – Welcher?**

**Seit wie vielen Monaten haben Sie die Symptome** (Schmerzen, Bewegungseinschränkungen…), **die zur Schienentherapie geführt haben?** (Vom ersten Auftreten der Symptome bis heute.)
_______ Monate

	Oberkiefer	Unterkiefer
**Ich habe eine Schiene im:**	○	○

Die folgenden Aussagen beziehen sich auf Ihre Zufriedenheit/Unzufriedenheit mit der Schiene.	Stimme völlig zu	Stimme eher zu	Stimme eher nicht zu	Stimme gar nicht zu
Meine Schiene ist unhygienisch.	○	○	○	○
Meine Schiene hat Mängel (z.B. Risse).	○	○	○	○
Meine Schiene sitzt nicht gut (z.B. zu stramm/drückt an Zähnen oder Zahnfleisch).	○	○	○	○
Meine Schiene ist aus einem Material gefertigt, das ich ungern in meinem Mund habe.	○	○	○	○
Die Schiene sieht optisch/ästhetisch nicht gut aus.	○	○	○	○
Ich empfinde die Schiene als zu dick/unangenehm geformt.	○	○	○	○
Der Geschmack der Schiene ist unangenehm.	○	○	○	○

In den folgenden Aussagen geht es um unerwünschte Nebeneffekte der Schiene.	Stimme völlig zu	Stimme eher zu	Stimme eher nicht zu	Stimme gar nicht zu
Das Tragen der Schiene beeinträchtigt meine Sprache.	○	○	○	○
Ich kann schlechter atmen, wenn ich die Schiene trage.	○	○	○	○
Mein Mund wird durch das Tragen der Schiene trocken.	○	○	○	○
Durch das Tragen der Schiene habe ich einen erhöhten Speichelfluss.	○	○	○	○
Ich sehe einen Zusammenhang zwischen dem Tragen der Schiene und Mundgeruch.	○	○	○	○
Ich kann schlechter schlafen, wenn ich die Schiene trage.	○	○	○	○
Ich fühle mich unwohl, wenn ich die Schiene trage.	○	○	○	○
**FALLS** Sie sich beim Tragen der Schiene unwohl fühlen: **Können Sie das Unwohlsein genauer beschreiben?**				

Weitere, nicht genannte Nebeneffekte:

**Was hat Ihr/e Zahnarzt/-ärztin empfohlen: Sollen Sie die Schiene beim Schlafen und/oder tagsüber tragen?**	
Ich soll die Schiene nur beim Schlafen tragen.	○
Ich soll die Schiene nur tagsüber tragen.	○
Ich soll die Schiene beim Schlafen <u>und</u> tagsüber tragen.	○
Ich soll die Schiene beim Schlafen <u>oder</u> tagsüber tragen.	○
Mein Zahnarzt/-ärztin hat mir diesbezüglich keine Empfehlungen gegeben.	○

Unabhängig davon, ob Sie Ihre Schiene tagsüber oder beim Schlafen tragen sollen…<br>**…was hat Ihr/e Zahnarzt/-ärztin empfohlen: Wie häufig sollen Sie Ihre Schiene tragen?**	
Ich soll die Schiene jeden Tag und/oder Nacht tragen.	○
Ich soll die Schiene in wöchentlichem Wechsel tragen und nicht tragen.<br>→ Falls *„tragen und nicht-tragen in wöchentlichem Wechsel"* empfohlen wurde:<br>Gelingt es Ihnen dies umzusetzen? ○ Ja ○ Nein	○
Ich soll die Schiene nur einige Male pro Woche tragen.<br>→ Anzahl der Tage, an denen ich die Schiene tragen soll:<br>_______ Tage (**max. 6**)	○
Ich soll die Schiene nur dann tragen, wenn Bedarf besteht.<br>Falls *„tragen bei Bedarf"* empfohlen wurde:<br>Gelingt es Ihnen dies umzusetzen? ○ Ja ○ Nein	○
Mein Zahnarzt/-ärztin hat mir diesbezüglich keine Empfehlung gegeben.	○

Im Folgenden geben Sie bitte an, wovon Ihr Trageverhalten **positiv** beeinflusst wird.
Bitte bewerten Sie, **ob** die genannten **Gründe** Sie dazu **motivieren** Ihre Schiene **häufiger** oder sogar **deutlich häufiger zu tragen**. Sie haben auch die Möglichkeit „Trifft nicht zu." anzukreuzen.

**Gründe** ↓	**Folgende Gründe motivieren mich, meine Schiene … zu tragen.**		
	**…deutlich häufiger…**	**…häufiger…**	**Trifft nicht zu.**
Weil mir das Tragen der Schiene sinnvoll erscheint.	○	○	○
Wegen der Erklärungen und Empfehlungen meines Zahnarztes.	○	○	○
Weil ich bereits seit langer Zeit (mindestens ein Jahr lang) Beschwerden habe.	○	○	○
Weil ich mir vorgenommen habe, die Schiene häufig zu tragen.	○	○	○
Weil die Schiene aktuelle Beschwerden lindern kann.	○	○	○
Weil die Schiene langfristig meine Beschwerden lindern wird.	○	○	○
Weil ich schon einen positiven Effekt spüre.	○	○	○
Weil ich (noch) keinen positiven Effekt spüre.	○	○	○
Weil ich Angst davor habe, dass ich ohne Schiene meine Zähne „wegknirsche"/viel Zahnhartsubstanz verliere.	○	○	○

Weitere nicht genannte Gründe, die dazu führen, dass ich die Schiene häufiger trage:

Im Folgenden geben Sie bitte an, wovon Ihr Trageverhalten **negativ** beeinflusst wird.
Bitte bewerten Sie, **ob** die genannten **Gründe** Sie dazu bewegen Ihre Schiene **seltener** oder sogar **deutlich seltener zu tragen**. Sie haben auch die Möglichkeit „Trifft nicht zu.“ anzukreuzen.

Gründe ↓	Folgende Gründe bewegen mich dazu, meine Schiene … zu tragen.		
	…deutlich seltener…	…seltener…	Trifft nicht zu.
Weil ich nur wenig Vertrauen in meinen Zahnarzt habe.	○	○	○
Wegen mangelnder Aufklärung zur Schienentherapie durch meinen Zahnarzt.	○	○	○
Weil ich es vergesse.	○	○	○
Weil ich gestresst bin.	○	○	○
Wegen Kummer/ Sorgen/ Lebensumständen, die gerade wichtiger erscheinen als das Tragen der Schiene.	○	○	○
Weil es mir unangenehm ist, die Schiene vor anderen Personen (z.B. PartnerIn/MitbewohnerIn/Arbeitskollege/in) zu tragen.	○	○	○
Weil ich ein Unwohlsein beim Tragen der Schiene empfinde.	○	○	○
Weil ich keine Symptome mehr habe (z.B. keine Schmerzen mehr).	○	○	○
Weil ich an dem Therapieziel (z.B. Schmerzfreiheit) Zweifel habe.	○	○	○
Weil ich mich (noch) nicht an die Schiene gewöhnt habe.	○	○	○
Weil meine Beschwerden sich durch die Schiene verschlimmern.	○	○	○
Weil ich noch keinen Behandlungseffekt spüre.	○	○	○
Weil ich bereits einen Behandlungseffekt spüre.	○	○	○
Weil ich zu sehr unterschiedlichen Zeiten zu Bett gehe.	○	○	○
Weil ich (auch ohne Schiene) Schlafprobleme habe.	○	○	○
Weil ich durch das Tragen der Schiene Schlafprobleme habe.	○	○	○
Wegen der schlechten Hygiene meiner Schiene.	○	○	○
Wegen Mängeln/Rissen an der Schiene.	○	○	○
Wegen des schlechten Sitzes der Schiene.	○	○	○
Wegen des schlechten Geschmacks der Schiene.	○	○	○
Wegen der störenden Oberfläche (z.B. Rauigkeit).	○	○	○
Wegen der schlechten Optik/Ästhetik der Schiene.	○	○	○
Wegen der störenden Form/Dicke der Schiene.	○	○	○
Weil die Schiene mich beim Sprechen beeinträchtigt.	○	○	○
Weil die Schiene mich beim Atmen beeinträchtigt.	○	○	○
Weil mein Mund durch die Schiene sehr trocken wird.	○	○	○
Weil mein Speichelfluss sich durch die Schiene verstärkt.	○	○	○
Weil die Schiene bei mir zu Mundgeruch führt.	○	○	○

Weitere nicht genannte Gründe, die mich davon abhalten die Schiene zu tragen:

UNIVERSITÄT LEIPZIG

Universitätsklinikum Leipzig
Medizin ist unsere Berufung.

Im Folgenden wird erfragt, an wen Sie sich bisher bezüglich Ihrer Beschwerden im Mund-/Gesichtsbereich gewandt haben. Bitte wählen Sie aus, was auf Sie zutrifft. Eine **Mehrfachauswahl** ist **möglich**.<br><br>**Welche Personengruppe(n) haben Sie bereits wegen Ihrer Beschwerden im Mund-/ Gesichtsbereich aufgesucht/kontaktiert?**	
Hals-Nasen-Ohren-Arzt	☐
Mund-Kiefer-Gesichtschirurg	☐
Orthopäde	☐
Physiotherapeut	☐
Anderer Zahnarzt/-ärztin als der-/diejenige, bei dem ich nun die Schiene bekommen habe	☐
Ich habe bisher deswegen keine andere Personengruppe aufgesucht/kontaktiert. (Neben meinem Zahnarzt/meiner Zahnärztin, der/die mir nun die Schiene eingegliedert hat.)	☐
Ich habe folgende weitere Personengruppe(n) deswegen kontaktiert:	☐

Schließlich benötigen wir für die Auswertung noch folgende Angaben zu Ihrer Person.			
**Ihr Geschlecht:**	◯ **männlich**	◯ **weiblich**	◯ **divers**
**Ihr Alter:**	**Jahre**		

**Welches ist Ihre <u>höchste Schulausbildung</u>?**	
Ohne allgemeinen Schulabschluss	○
Noch in schulischer Ausbildung	○
Haupt-/Volksschulabschluss	○
Mittlerer Abschluss, Abschluss der polytechnischen Oberschule oder gleichwertiger Abschluss	○
Fachhochschul- oder Hochschulreife	○

**Wie viele Schienen hatten Sie bereits/ oder ist dies Ihre erste Schiene?**	
◯	Dies ist meine erste Schiene.
◯	Ich hatte bereits eine/ mehrere Schienen. Anzahl vorheriger Schienen: _____

Herzlichen Dank für Ihre wertvolle Mitarbeit und das Vertrauen!
