# Supplementary material for: Factors Influencing Adherence to Therapy With Occlusal Splints—A Multicentre Questionnaire Based Study
Source: J Oral Rehabil. 2025 Jul 24;52(12):2209–18. doi: 10.1111/joor.70023 (PMC12624160; doi:10.1111/joor.70023)
Supplement: Supplementary file 2 — Table S1. Dissatisfaction variables and their frequencies (N = 200). Table S2. Rare mentioned side effects listed in alphabetic order. [file JOOR-52-2209-s002.docx]

## Supplementary Tables

**Supplementary Table S1** Dissatisfaction variables and their frequencies (N=200)

**Supplementary Table S2** Rare mentioned side effects listed in alphabetic order

**Supplementary File S3** Questionnaire

**Supplementary Table S1** Dissatisfaction variables and their frequencies (N=200)

|  | Low adherence | High adherence | No indication for this factor |
| --- | --- | --- | --- |
| Unhygienic | 2 | 3 | 195 |
| Defects | 4 | 11 | 185 |
| Poor fit | 14 | 6 | 180 |
| Unwanted Material | 16 | 9 | 175 |
| Poor Aesthetics | 16 | 14 | 170 |
| Too thick/uncomfortable shape | 23 | 14 | 163 |
| Unpleasant taste | 14 | 6 | 180 |

**Supplementary Table S2**  Rare mentioned side effects listed in alphabetic order

| Additional work due to cleaning the splint |
| --- |
| “Different bite in the morning” |
| Difficulty breathing |
| Dry lips |
| (Falling) asleep problems |
| Feeling of pressure on teeth |
| Gag reflex |
| Noticeable foreign object |
| Open mouth with sialorrhea |
| Stronger grinding than without splint |
| Stronger snoring |
| Unconscious removal/falling out of the splint at night |
